# Assessment of immunomodulatory activity of aqueous extract of *Calamus rotang*

**Published:** 2017

**Authors:** Amit Gupta, Sushama Ravindra Chaphalkar

**Affiliations:** *Vidya Pratishthan’s School of Biotechnology (VSBT, Research centre affiliated to Savitribai Phule Pune University) Baramati, Maharashtra, India*

**Keywords:** Calamus rotang, Immunomodulatory, Aqueous extract, PBMC, Hepatitis B vaccine

## Abstract

**Objective:**

There are a number of medicinal plant products which has been used to treat various immunological diseases. Out of these medicinal plants, *Calamus rotang* has shown several medicinal properties including anti-viral, anti-diabetic, anti-inflammatory effects. Normally, the roots of *C. rotang* are used in various ailments to cure piles, burning sensation, cough, leprosy and bleeding disorder and also it was used in the treatment of inflammation. In the present study, our group were investigated the immunomodulatory activity of aqueous extract of *C. rotang* root in human whole blood and peripheral blood mononuclear cells (PBMC) using hepatitis B vaccine (HBsAg) as an antigen.

**Materials and Methods:**

Variable doses of root aqueous extract (0.5 – 30 mg/ml, 100 µl) was administered to human whole blood and PBMC using hepatitis B vaccine containing surface antigen (HBsAg; 20 µg/ml, 10 µl) as specific antigen in order to estimate the total blood counts in human whole blood and nitric oxide production and CD14 FITC surface marker from human PBMC.

**Results:**

Overall, the results showed that roots aqueous extract of *C. rotang *showed remarkable increase in the number of blood counts in human whole blood at lower doses (0.5 mg/ml). In addition, root aqueous extract of *C. rotang* also showed the same pattern in case of nitric oxide production and estimation of CD14 FITC surface marker in human PBMC.

**Conclusion:**

Altogether, the results suggest that root aqueous extract of *C. rotang* showed immunomodulatory activity.

## Introduction

In various investigations on medicinal plant products against specific and non-specific antigens, several immunopharmacological activities such as immunomodulatory, anti-inflammatory (He and Dai, 2011 ▶), anti-viral (Barrett et al., 1999) and anti-diabetic (Sujith et al, 2011 ▶) effects were shown. These medicinal plant products have been reported to be used for various immunological diseases or disorders (e.g. rheumatoid arthritis) (Aletaha et al., 2010 ▶). The capacity or ability of these medicinal plants was particularly related to inhibition or stimulation of B and T cell immune response with respect to immune-mediated disorders (Mihich and Ehrke, 2000 ▶; Gupta and Chaphalkar, 2015 ▶). According to Ayurveda, millions of medicinal plants are present all over the world and most of them possess various medicinal properties (Gupta et al., 2014 ▶). Till now, most of the medicinal plants have not investigated and these plants should be used for treatments of various diseases or disorders.

*Calamus rotang *(commonly known as vet), a medicinal plant which belong to the family *Arecaceae*, is generally found in Southern and central parts of India (i.e. Maharashtra, Andhra Pradesh, etc.). These medicinal plant products possess several medicinal uses because of the presence of secondary metabolites like saponin in the stem, alkaloid in the leaves and flavonoid in the root (Soladoye and Chukwuma, 2012 ▶; Ripa et al., 2015 ▶]. Information on medicinal plant products and their medicinal uses have inspired us to investigate the immunomodulatory effect of root aqueous extract of *C. rotang* on total blood counts in human whole blood and estimate the nitric oxide production and CD14 FITC surface marker in human PBMC. Therefore, this immunopharmacological study was conducted to determine the immunomodulatory activity of *C. rotang*.

## Materials and Methods

**Collection and preparation of plant material (Udyan, Baramati**)

Fresh roots of plant material (*C. rotang*) were collected from the garden of Vidya Pratishthan’s School of Biotechnology, Vidyanagari, MIDC road, Baramati, District Pune. The fresh roots of *C. rotang* were macerated with liquid nitrogen using mortar and pestle and then, filtered through a piece of clean Whatman filter paper. The freshly prepared root aqueous extract of *C. rotang* was transferred to a 50 ml tube for further immunopharmacological studies.

**Qualitative analysis of secondary metabolites**

The root aqueous extract of *C. rotang* was subjected to qualitative chemical screening for the identification of various secondary metabolites. The analysis of phytoconstituents of *C. rotang* root indicated the presence of flavonoids (alkaline reagent test), steroids/triterpenoids (acetic anhydride test) and glycosides (Borntranger test).

**Determination of total blood counts using flow cytometer**

In order to determine the immunomodulatory effect of aqueous root extract of *C. rotang* in human whole blood and PBMC. These blood samples (anti-coagulant EDTA, non-infected) were collected from Mangal Pathology Laboratory, Maharashtra, India and analyzed or processed at the VSBT, Baramati, Maharashtra, India, between November 2014 and January 2015. 

Flow cytometry has long been used in various immunopharmacological studies and provides valuable information within minutes of analysis or acquisition. To understand the principle as well as concept of flow cytometer; variable doses of aqueous root extract (0.5 – 30 mg/ml, 100 µl) and HBsAg (20 µg/ml, 10 µl) were administered to human whole blood (100 µl) for 2 hr incubation (carbon dioxide incubator, 37 C and 5 % CO_2_) in order to estimate total blood counts using forward scatter (FSC, shape and size) and side scatter (granularity of the cell). After incubation, blood samples were lysed with FACS lysing solution and washed two times with phosphate buffered saline. Finally, these samples were analyzed through flow cytometer using forward and side scatter (FACS Calibur). The flow cytometer gating was applied for data acquisition of 10000 events of cell populations representing different phenotypes analyzed using cell quest software (Gupta and Chaphalkar, 2015 ▶).

**Estimation of nitric oxide production and CD14 FITC monocyte surface marker from human PBMC**

Briefly, PBMC were extracted from human whole blood by means of density gradient centrifugation. In this experiment, PBMC (10^6^ cells/ml, 100 µl) were plated in 96-well plates along with HBsAg (20 µg/ml, 10 µl) antigen and variable doses of aqueous root extract of *C. rotang* (0.5 – 30 mg/ml, 100 µl) and incubated for 24 hr . PBMC cells containing media (100 µl) were used as control. After incubation and centrifugation, the supernatant was collected for the estimation of nitric oxide and those cells settled at the bottom were used for the estimation of CD14 monocyte surface marker using flow cytometer as discussed above.

The quantity of nitrite accumulated in the cell culture supernatant of PBMC treated with root aqueous extract was measured and considered as an indicator of nitric oxide production. Briefly, 50 μl of cell culture supernatant of PBMC along with root aqueous extract of *C. rotang* was mixed with 50 μl of Griess reagent (1% sulfanilamide and 0.1% naphthylethylenediaminedihydrochloride in 2- 2.5% phosphoric acid) and 96-well plates were incubated at room temperature for 7-8 min. Then, absorbance or optical density was measured at 540 nm using a microplate reader. The cell culture medium (PBS, phosphate buffered saline containing 10 % fetal bovine serum) was used as blank. The nitrite quantity (µM) was determined based on a sodium nitrite standard curve (Gupta et al, 2014 ▶). 

**Statistical analysis**

Data are reported as means ± standard error of mean (SEM). The difference between the control and treated groups is determined by One way ANOVA test (Bonferroni multiple comparison test) and p<0.05 was considered as statistically significant.

## Results

**Estimation of total blood counts using flow cytometer**

**Figure 1:**
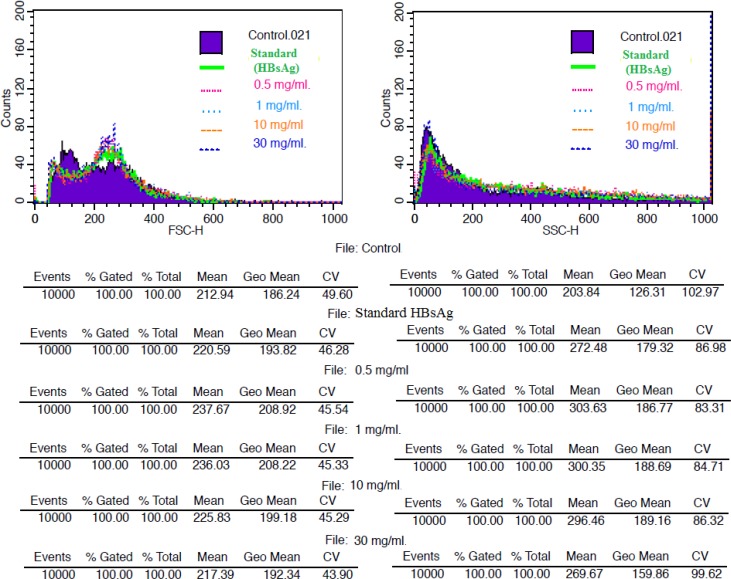
Effect of root aqueous extract of *Calamus* *rotang* on human whole blood using flow cytometry (Forward scatter (FSC) and side scatter (SSC)). Data acquisition of 10000 events and fraction or separation of cell populations representing forward and side scatter using cellquest software

The total blood counts in human whole blood treated with variable doses of root aqueous extract of *C. rotang* were measured using flow cytometry (Figure 1). The flow cytometric results showed that the aqueous root extract (0.5 mg/ml) increased the total blood counts (with respect to enhancement of forward scatter and side scatter) as compared to control and standard. HBsAg used as standard for these studies and the results also showed an increase in blood counts as compared to control. 

**Estimation of nitric oxide production and CD14 FITC surface marker by flow cytometry**

**Figure 2:**
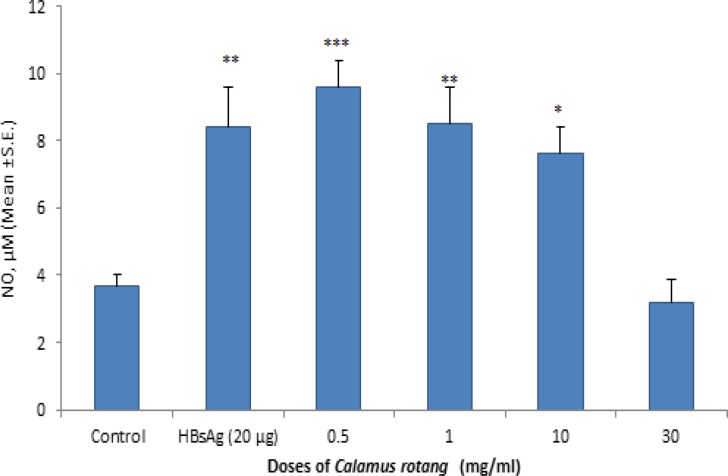
Estimation of nitric oxide (NO) production**.** The supernatant nitrite concentration was determined by Griess reagent after 24 hr of culture of PBMC in the presence of root aqueous extract of *Calamus* *rotang*. Values are expressed as Means ± SEM. The difference between the control and treated groups is determined by One way ANOVA test (Bonferroni multiple comparison test). *p<0.05; **p<0.01; ***p<0.001

**Figure 3:**
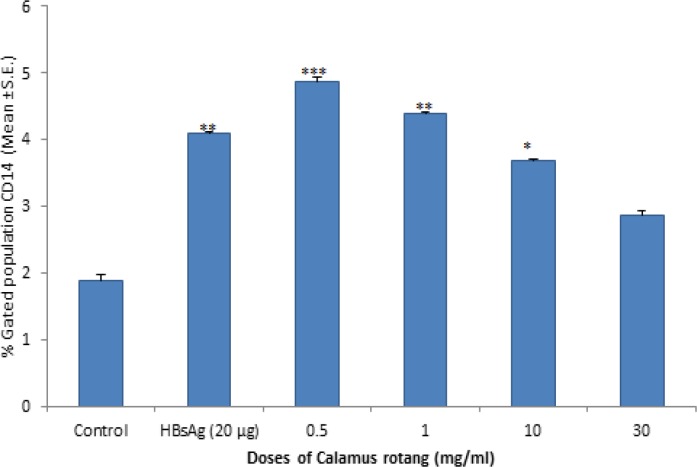
Estimation of CD14 surface marker on human peripheral blood mononuclear cells (PBMC) using flow cytometry. Cells were stained with CD14 FITC surface marker and then, lysed and washed in PBS and analyzed using a flow cytometer (FACS Calibur). Values are expressed as Mean ± SEM. The difference between the control and treated groups is determined by One way ANOVA test (Bonferroni multiple comparison test). *p<0.05; **p<0.01; ***p<0.001

The effect of aqueous root extract of *C. rotang* in cell culture supernatant of PBMC for the estimation of nitric oxide abd CD14 FITC surface marker was shown in Figure 2 and 3. The results showed that root aqueous extract (at lower doses i.e. 0.5 mg/ml) and HBsAg increased nitric oxide production and CD14 FITC surface marker as compared to control. CD14 is a marker which is present on the surface of monocytes. Overall, the results suggest that root aqueous extract showed immunomodulatory activity at lower doses with respect to enhancement of nitric oxide production and CD14 FITC surface marker.

## Discussion

The immune system is involved in the etiology as well as pathophysiological mechanisms of many diseases (e.g. bacteria, viruses, protozoan infections). The modulation of the immune responses using different medicinal plants in order to reduce the burden of various human diseases, has been of immense interest for many researchers. Generally, these medicinal plant products (i.e. leaves, root, stem, seed, fruit, etc.) are rich sources of various primary and secondary metabolites (flavonoids, terpenoids, alkaloids, etc.) (Gupta et al., 2014 ▶). These metabolites provide alternative treatments for various human diseases (e.g. viral and bacterial infections) and also possess beneficial therapeutic properties including anti-oxidant, anti-inflammatory, anti-microbial and immunomodulatory effects (Gupta et al., 2015 ▶; Khajuria et al., 2007 ▶; Davis and Kuttan, 2007 ▶). Some of medicinal plants (e.g. *Picrorhiza kurroa *(Gupta et al., 2015 ▶), *Boswellia serrata *(Khajuria et al., 2007 ▶), *Withania sominiferra *(Davis and Kuttan, 2007 ▶)) have shown immunomodulatory activities. Out of these medicinal plants, here we showed that *C. rotang* has immunomodulatory effects.

In the present study, *C. rotang* (root aqueous extract) showed an immunomodulatory effect against HBsAg at lower doses in human whole blood (total blood counts) and PBMC (nitric oxide production and CD14 FITC monocyte surface marker). This stimulatory effect at lower doses of the aqueous root extract is due to the presence of secondary metabolites which can augment cell-mediated immune response by stimulating these parameters as mentioned above. 

In order to validate the potential effect of root aqueous extract of *C. rotang* on human PBMC nitric oxide production; this parameter was used as an immunopharmacological indicator in preliminary screening for the determination of immunomodulatory effects.

Venous blood was collected from the healthy volunteers (samples received from Mangal pathology lab). PBMCs were separated by means of gradient centrifugation as it is suggested that the PBMC provides relatively accurate as well as reliable information regarding immunopharmacological activity of treated as well as control samples in human immune system (Wright et al., 1990 ▶; Ziegler-Heitbrock, 1995 ▶). The main advantage of this initial preliminary screening is its sensitivity, general feasibility, low cost and possibility of large-scale performance. These preliminary screening tests (i.e. nitric oxide production (using Elisa method) and estimation of CD14 monocyte surface marker (using flow cytometery) from human PBMC are much more sensitive with respect to the activation signal of LPS, and should always be accompanied by careful examination of the aqueous extract samples for possible contamination with LPS (Wright et al., 1990 ▶; Ziegler-Heitbrock, 1995 ▶). It has been suggested that production of nitric oxide from macrophages, dendritic cells and PBMC may depend on cell types and their species origin, as different cells of our immune system have obviously different requirements for signal transduction pathways (Wright et al., 1990 ▶; Ziegler-Heitbrock, 1995 ▶). On the other hand, CD14 (55 kDa, glycoprotein with multiple leucine rich repeats) surface marker is normally present on monocyte and macrophages and serves as homing receptor for the lipopolysaccharide (LPS) of gram negative bacteria (Wright et al., 1990 ▶; Ziegler-Heitbrock, 1995 ▶; Haziot et al., 1995 ▶). In this study, the results suggests that roots aqueous extract of *C. rotang* showed increased in nitric oxide production and CD14 FITC surface marker by human PBMC. In addition, it also showed the same pattern in case of total blood counts. At lower doses, *C. rotang* roots aqueous extract increased the total blood counts as compared to control. Overall, the results suggest that the roots aqueous extract of *C. rotang* has immunomodulatory effect.

In the present study, *C. rotang* significantly stimulated the production of nitric oxide and CD14 FITC monocyte surface marker in human PBMC and also increase in total blood counts in human whole blood. Further investigations on root aqueous extract of *C. rotang* should focus on the *in vivo* assessment of its various immunopharmacological activities and also should identify the major active components responsible for *C. rotang* immunomodulatory activities.
